# The Beat

**DOI:** 10.1289/ehp.119-a512b

**Published:** 2011-12-01

**Authors:** Erin E. Dooley

## Scientists Investigate Burned Gulf Spill Emissions

Much of the oil spilled during the BP *Deepwater Horizon* disaster in the Gulf of Mexico was burned to keep it from reaching the shoreline or harming sea life. NOAA scientists have analyzed the gas and aerosol emissions resulting from the burning of the spilled oil and found that over a 9-week period more than 1 million pounds of black carbon were generated, roughly the amount emitted in the same period by ship traffic in the region.^1^ Compared with ship emissions, the particles generated by the oil burning rose to higher altitudes but also were larger and attracted fewer other substances, which may shorten their lifetime and make them less of a health and climate threat. These findings could be used by decisionmakers to help assess the tradeoffs of various response strategies during future disasters.

**Figure:**
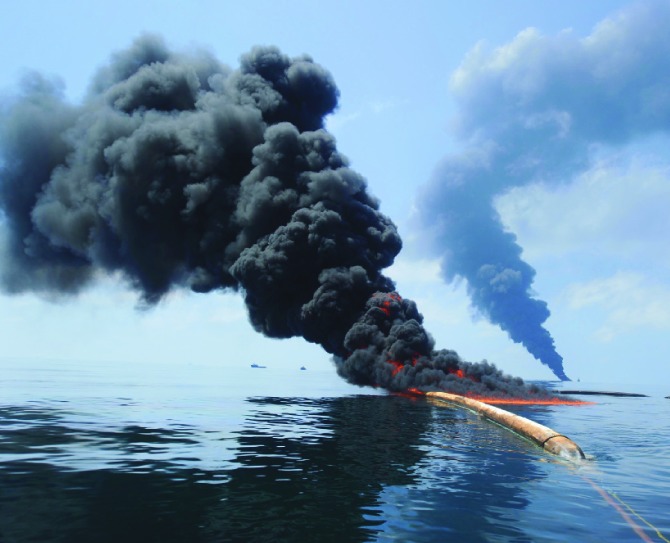
Oil from the BP Deepwater Horizon disaster during a controlled burn, 6 May 2010. Justin E. Stumberg/U.S. Navy via Getty Images

## Norovirus Persists in Groundwater

A new study has shown that norovirus in groundwater can remain infectious for an unexpectedly long time—at least 61 days.^2^ With symptoms that include diarrhea and vomiting, norovirus causes more than 20 million cases of gastroenteritis in the United States each year, according to CDC estimates.^3^ The virus, which can enter groundwater via leaky sewer pipes or septic tanks, is best removed from drinking water using nanofiltration, reverse osmosis, distillation, or ultraviolet treatment; chemical treatment is only moderately effective.^4^

**Figure:**
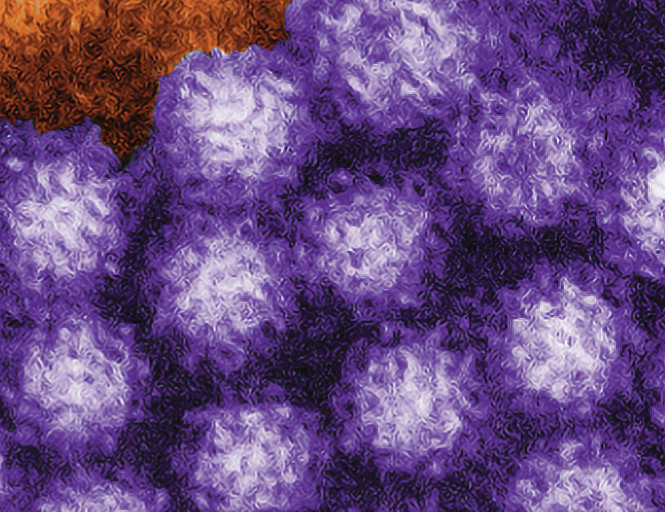
Transmission electron micrograph of norovirus particles. Charles D. Humphrey / Centers for Disease Control and Prevention

## NNI Updates Nanotechnology Research Strategy

The multiagency National Nanotechnology Initiative has released an updated strategy document that identifies key environmental, health, and safety research needs in regards to engineered nanomaterials (ENMs) and the products that contain them.^5^ The document focuses on six core research categories to help guide the responsible development of nanotechnology: nanomaterial measurement tools and protocols, human exposure assessment, human health, environmental fate and impact, risk assessment and risk management, and informatics and modeling. The emphasis on informatics and modeling, new for this update of the strategy, reflects the need for a way to organize the rapidly growing wealth of data on ENMs.

## Help for Disease-Stricken Coral Reefs?

Coral reefs sequester carbon dioxide, support fisheries, protect the coastline from storms, and help generate tourism revenue. But reefs around the world are being compromised by changing ocean waters and further threatened by opportunistic pathogens such as *Serratia marcescens*, which causes white pox in Caribbean corals. Researchers have discovered that a cocktail of other bacteria isolated from Caribbean reef tracts, when administered under laboratory conditions, helped prevent white pox disease progression in the polyp *Aiptasia pallida*, a coral cousin and surrogate model for coral research.^6^ The researchers believe it may be possible to use beneficial probiotic-like bacteria as a tool for the proactive management of coral reefs.

## EPA Announces Final Plan to Assess Fracking Impact on Water

After months of public meetings and a review by the agency’s independent Science Advisory Board, the EPA recently announced its final research plan for hydraulic fracturing (“fracking”).^7^ The study plan encompasses the full cycle of how water is acquired, used, and disposed of during fracking. The initial research results and study findings will be released in 2012, with a final report expected in 2014.

**Figure:**
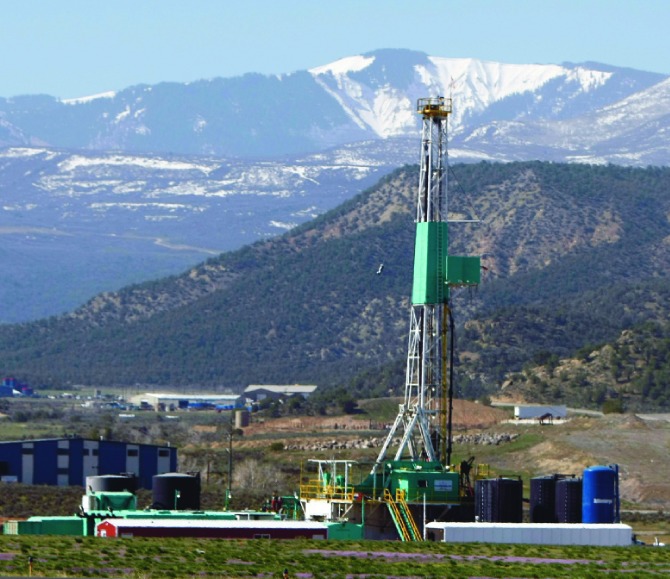
A natural gas rig near Rifle, Colorado. © AP Photo/David Zalubowsk
